# Self-management including exercise, education and activity modification compared to usual care for adolescents with Osgood-Schlatter (the SOGOOD trial): protocol of a randomized controlled superiority trial

**DOI:** 10.1186/s13102-024-00870-0

**Published:** 2024-04-20

**Authors:** Kasper Krommes, Kristian Thorborg, Mikkel Bek Clausen, Michael Skovdal Rathleff, Jens Lykkegaard Olesen, Thomas Kallemose, Per Hölmich

**Affiliations:** 1 Sports Orthopedic Research Center – Copenhagen (SORC-C), Department of Orthopedic Surgery, Amager-Hvidovre, Copenhagen University Hospital, Kettegaard Allé 30, Hvidovre, DK-2650 Denmark; 2 Department of Clinical Medicine, University of Copenhagen, Blegdamsvej 3B, DK-2200 Copenhagen N, Denmark; 3 Department of Midwifery, Physiotherapy, Occupational Therapy and Psychomotor Therapy, Faculty of Health, University College Copenhagen, Sigurdsgade 26, DK-2200 Copenhagen N, Denmark; 4 Center for General Practice at Aalborg University, Aalborg. Fyrkildevej 7, DK-9220 Aalborg, Denmark; 5 Department of Health Science and Technology, Aalborg University, Selma Lagerløfs Vej 249, DK-9220 Aalborg, Denmark; 6 Department of Clinical Research, Amager-Hvidovre, Copenhagen University Hospital, Kettegaard Alle 30, Hvidovre, DK-2610 Danmark

**Keywords:** Osgood-Schlatter, Apophysitis, Adolescents, Sport, Physical activity, Knee, Ultrasound, Conservative treatment, Activity modification, Self-management, Load management

## Abstract

**Background:**

Osgood-Schlatter is the most frequent growth-related injury affecting about 10% of physically active adolescents. It can cause long-term pain and limitations in sports and physical activity, with potential sequela well into adulthood. The management of Osgood-Schlatter is very heterogeneous. Recent systematic reviews have found low level evidence for surgical intervention and injection therapies, and an absence of studies on conservative management. Recently, a novel self-management approach with exercise, education, and activity modification, demonstrated favorable outcomes for adolescents with patellofemoral pain and Osgood-Schlatter in prospective cohort studies.

**Aim:**

The aim of this trial is to assess the effectiveness of the novel self-management approach compared to usual care in improving self-reported knee-related function in sport (measured using the KOOS-child ‘Sport/play’ subscale) after a 5-month period.

**Methods:**

This trial is a pragmatic, assessor-blinded, randomized controlled trial with a two-group parallel arm design, including participants aged 10–16 years diagnosed with Osgood-Schlatter. Participants will receive 3 months of treatment, consisting of either usual care or the self-management approach including exercise, education, and activity modification, followed by 2 months of self-management. Primary endpoint is the KOOS-child ‘Sport/play’ score at 5 months. This protocol details the planned methods and procedures.

**Discussion:**

The novel approach has already shown promise in previous cohort studies. This trial will potentially provide much-needed level 1 evidence for the effectiveness of the self-management approach, representing a crucial step towards addressing the long-term pain and limitations associated with Osgood-Schlatter.

**Trial registration:**

Clinicaltrials.gov: NCT05174182. Prospectively registered December 30th 2021. Date of first recruitment: January 3rd 2022. Target sample size: 130 participants.

## Introduction

### Background and rationale

Osgood-Schlatter affects up to 1 in 10 physically active adolescents and is the most common growth-related injury [1, 2]. The condition affects the knee, specifically the proximal tibial apophysis [3]. Osgood-Schlatter can lead to long-term pain and swelling, decreased quality of life, and limit participation in sports and physical activity [4–7]. Even years after diagnosis, adolescents can experience continued pain, discontinuation of sports-participation, impairments in knee function, and sonographic changes can persist [5–9]. Recent systematic reviews identified only low level evidence for surgical intervention and injection therapies for recalcitrant cases, and a complete absence of studies on conservative management [10, 11]. The recommended types of conservative management modalities for Osgood-Schlatter is abundant and conflicting in the literature [10, 12–14]. The same is evident in studies of clinical practice, in which clinicians and patients report a range of different advice and modalities utilized [15, 16]. This reflects the lack of evidence regarding this area for first-line conservative treatments, which leaves clinicians to an experience-based approach to management of Osgood-Schlatter. This highlights the need for an effective evidence-based conservative management approach. In 2020, a promising cohort study was published by Rathleff et al. where adolescents with Osgood-Schlatter received a self-management approach containing gradual exposure to sports and physical activity using a guidance tool based on pain response, and progressive exercise therapy. This was associated with successful outcomes, such as rating of change, pain, and sports participation [17].

### Review of the literature

Two recent systematic reviews on treatment for adolescents with Osgood-Schlatter found no studies assessing conservative treatments [10, 11]. Three studies were identified assessing surgical or injection-based interventions; all exhibited significant methodological flaws, such as selective or shifting of outcomes reported, and lack of blinding of participants and personnel [11]. The two reviews concluded that there is a paucity of evidence to guide clinical practice, and a need for trials on conservative management [11]. At this point in time four trials on Osgood-Schlatter are registered on clinicaltrials.gov; 1 trial comparing static stretching vs. active elongation (estimated completion November 2013); 1 trial comparing cast immobilization vs. advice to rest (recruitment completed December 2016); 1 trial comparing myofascial massage vs. usual care (recruitment completed September 2022); and 1 recent trial from a member of our study group comparing some of the different components in the experimental intervention in a 3-armed trial, thus comparing tailored progressive loading and return to sport vs. pain guided activity vs. 4 week rest (recruitment started January 2023). The need for this trial therefore remains.

### Choice of comparators

An international study among 251 practitioners with a special interest in Osgood-Schlatter found that, besides patient education and exercise therapy, treatment approaches are highly heterogenous [15]. In lack of existing knowledge about usual care in the Danish healthcare system, and an absence of an established treatment approach in the literature, we have performed a step-wise sub-study to investigate current standard of care in Denmark, in order to compile a standardized treatment package as comparator [16]. In Denmark, clinicians who commonly see patients with Osgood-Schlatter disease are Sports Physiotherapists and general practitioners, mainly in private or primary practice, and Orthopedic Surgeons from secondary care orthopedic departments. Among these professions, we conducted interviews with 10 clinicians and surveyed 63 more, asking them about the modalities and advice they most frequently use when treating patients with Osgood-Schlatter [16]. Results were then combined with reports from patients seen in our outpatient clinic (*n* = 34) who were questioned in detail on what modalities and advice they had previously received [16]. The resulting list of modalities ordered by frequency where then combined and developed into a standardized treatment package with an accompanying patient-aimed leaflet [16]. An overview of the two treatments and their timing can be seen in Table 1.

**Table 1:** Short-form overview of the two treatments

	Experimental	Comparator
Month 0–1	Phase 1 • Break from sports and moderate-to-vigorous physical activity • Daily high-volume low load isometric training • High-load hip-abductor training every other day • Introduction to a pain-model for progression of exercises and exposure to sport/moderate-to-vigorous physical activity • Education on pain science and management	• Introduction of progressive balance and alignment exercises every other day and continued • Introduction of daily progressive quadriceps stretching • Advice on approaches for preventing/treating pain flares: • Cryotherapy after activity if painful • Sports taping • Handout and instructions in using a patella strap • Advice on potential prognosis • Advice participation in sports and physical activity when experiencing pain
Months 2–3	Phase 2 • Self-managed introduction of gradual exposure to sport based on the pain-model • Once acceptable sport-level achieved, self-managed gradual exposure to vigorous physical activity is introduced •Introduction of progressively more loaded isometric and subsequent dynamic weight-bearing exercises for the knee extensors • Continued high-load hip-abductor training every other day	
Months 4–5	Self-management phase • Complete self-management of pain vs. loading from participation in sports and physical activity • Potential self-management of self-chosen exercise dose	Self-management phase • Complete self-management of pain vs. loading from participation in sports and physical activity • Potential self-management of self-chosen exercise dose

### Aims and primary hypothesis

The primary aim is to investigate whether a self-management approach is superior to usual care in adolescents with Osgood-Schlatter. The primary endpoint is changes in self-reported function in sport from baseline to 5 months. A secondary aim is to investigate changes in secondary outcomes (acceptable symptoms state, frequency of pain flares, pain intensity, sports participation, sonographic severity, pain during knee-loading, rating of change), also at secondary timepoints (weekly from week 0 to 22, and at months 1, 2, and 3). Results on the primary and secondary aims and outcomes, are planned to be included in the primary report. The primary hypothesis, is that the novel self-management approach is superior to usual care after 5 months on improvements in self-reported function in sport, measured on the KOOS-child ‘Sport/play’ subscale, in adolescents with Osgood-Schlatter.

## Methods

### Trial design

The trial is an assessor-blinded randomized controlled superiority trial, with a two-group parallel arm design and 1:1 group allocation ratio. Besides assessor-blinding, we will blind participants and treatment-personnel to the superiority hypothesis and specific contents of the intervention they are not receiving/delivering. This protocol is structured according to the SPIRIT guidelines (Standard Protocol Items: Recommendations for Interventional Trials) [18]. We chose a superiority framework for two reasons; firstly, due to a preliminary study of the intervention showing promising results for patients having tried other treatments and with a significant duration of symptoms; and secondly, as the current usual care given is based on clinical expertise and experience [15, 16]. We found our design to be mostly pragmatic on the Pragmatic-Explanatory Continuum in terms of clinical domains, making the potential results fit for clinical implementation, and the trial will therefore provide evidence on the effectiveness of the intervention [19, 20].

#### Registration and reporting

The reporting of the results will adhere to the Consolidated Standards of Reporting Trials (CONSORT) [21]. The interventions are described according to current best-practice standards [22–24]. We have posted a previous version (v1.4) of this protocol and the statistical analysis plan to the ClinicalTrials.gov repository before starting recruitment as a supplementary to the registration (NCT05174182). The embedded statistical analysis plan follows regulatory and academic recommendations and guidelines, as well as open-science practices [25–28]. Exercises in the experimental intervention is described by parameters proposed by Toigo & Boutellier [24], and domains from CERT [23] (Checklist for Exercise Reporting Template) and TIDieR [22] (Template for Intervention Description and Replication).

#### Pilot study: results and amendments

The initial 15 participants acted as pilot-participants. The purpose of the internal pilot [29] was to assess the feasibility of the larger extensive full-scale study, to avoid any adverse consequences or unforeseen pitfalls during the main trial, and ensure that all trial components worked together [30, 31]. This did not incur any additional burden to participants or change their experience or procedures, compared to the intended setup for subsequent participants. Trial personnel recorded extra data during months 0–3 for these participants on specific pilot objectives. We prespecified pilot objectives and criteria for either a success or failure of the pilot study. If the aim and features of the pilot trial is aligned with the main trial and participant data are deemed compatible such that none or minimal amendments are needed for the main trial, their data be included in the analysis of the main trial [30]. The internal pilot were deemed as successful per pre-specified criteria, as only minor changes to trial-procedures were needed, and participant-data was considered compatible with inclusion in the main trial dataset [29, 30]. The following minor changes to procedures was implemented as a result of the internal pilot:

- Changes to the REDCap-project, such as removing obsolete fields, fixed validation values, change wrong field values.
- Changed sequence of some clinical measures to increase time-efficiency
- As one participant performed fewer than the planned 3 attempts of maximal knee extension strength test due to knee pain, the analysis of pain during testing will now consider the most painful attempt during these trials, rather a mean measure of pain across trials.
- Four participants experiences (minor) incidents of sensor-adhesive causing skin irritation. We changes the procedures for preventing and handling irritated skin to be more rigorous.

#### Embedded qualitative study

A qualitative study will be nested in the trial to understand barriers and facilitators to adhering to the interventions and describe the participant-perspective of undergoing the interventions as a whole [32]. This will support potential implementation and adjustments of the intervention. The interviews will be performed in groups stratified by group allocation and age, across 4–6 sessions in total. Participants from the main trial will be invited to group-sessions after undergoing their visit at 5 months (primary endpoint assessment). They will be provided with a leaflet detailing how the session will take place and what themes are planned. As compensation, participants will receive a voucher for movie theaters (280 dkr. ~ 38 EUR) as well as generous refreshments during the session. The sessions will take place at Hvidovre Hospital in a standard meeting room. An adjacent room with refreshments will be made available for parents. The sessions are planned for 2 × 45 min. Separated by a 45 min. break. As the population are young adolescents, the interviews will be performed as group-interviews to facilitate dialogue and discussions between participants, hopefully inviting participants to openly share their experiences in a safe space, motivated by a mostly shared experience. The planned sample size is based on field-specific guidance [33]. If sufficient Information Power does not appear to be achieved following this pre-planned number of interviews, more interviews will be conducted to reach sufficient Information Power. The sample size of *n* = 16 is based on the following elements:

- Aim: The aim of the study is not narrow or specifically around any one or few theoretical constructs. This adds to a larger sample size needed.
- Specificity: The sample of participants will be quite homogenous with one strata to (group allocation). This aspect will decrease the sample size needed.
- Theory: Our group will apply a theoretical framework to the design and analysis (theoretical domains and component constructs from the field of behavior-change) [34, 35]. These specifications will narrow the scope of data collection and analysis and will thus decrease the number of participants needed.
- Quality of dialogue: As the researcher conducting the interviews will have no experience, the quality of dialogue will be weak. However, an experienced qualitative researcher will supervise the first few interviews to facilitate a stronger quality of dialogue. Moreover, the interviewing researcher will have extensive knowledge of the subject matter. We therefore expect this aspect will neither require us to increase or lower the needed sample size.
- Our analysis will focus on creating understanding across participants with diverse experiences, not just for singular participants. This will increase the needed sample size.

Data will be organized using data derived coding, based on relevance to research questions and the theoretical constructs. Codes will be collapsed into categories and underlying themes based on prevalence of similarities and differences either within respondents or across respondents. Major themes can then be built upon other themes and coding. We will utilize an iterative approach to determine themes and overall interpretations, in line with the epistemology of circular deductive reasoning. Based on known theoretical constructs of behavior change [34, 35], we have used theory to comprise the interview-guide within the scope of the research questions. One specific study provides some evidence for choosing which behavior-change constructs to include in our interview guide. The study is a previous investigation of adolescents experiencing non-traumatic anterior knee pain, on how they achieve successful self-management [36]. This source, combined with clinical experience, has guided the choice and prioritization of domains and constructs.

### Setting and personnel

All trial-related procedures will take place at Hvidovre Hospital, Capital Region, Denmark, at either the Department of Orthopedic Surgery or Department of Physiotherapy. Intervention personnel will be 8–10 physiotherapists. Outcome assessors will be 3–4 physiotherapists. Personnel responsible for diagnosis, inclusion, end-of-study visit will be one physiotherapist (KK) and a potential trained replacement, who will not be blinded to either group allocation or outcome measures. The Medical Advisor will be a Chief Orthopedic Surgeon (PH), who will examine participants in need of a second opinion regarding initial diagnosis, AEs, or other sudden health deterioration in participants. A biostatistician (TK) blinded to group allocation in the dataset will perform the analyses.

### Interventions and implementation

#### Experimental intervention: self-management and activity modification

Based on previous work by Rathleff et al. for adolescents with patellofemoral pain and Osgood-Schlatter [17, 37], the experimental intervention will contain a self-management approach to activity modification of sports and physical activity, and progressive exercise therapy, delivered through 4 one-on-one visits lasting approximately 20 minutes (at months 0, 1, 2, 3) over 3 months with a physiotherapist and an accompanying leaflet with written and illustrated exercise description, self-management tools, and advice and information.

**Fig. 1:**
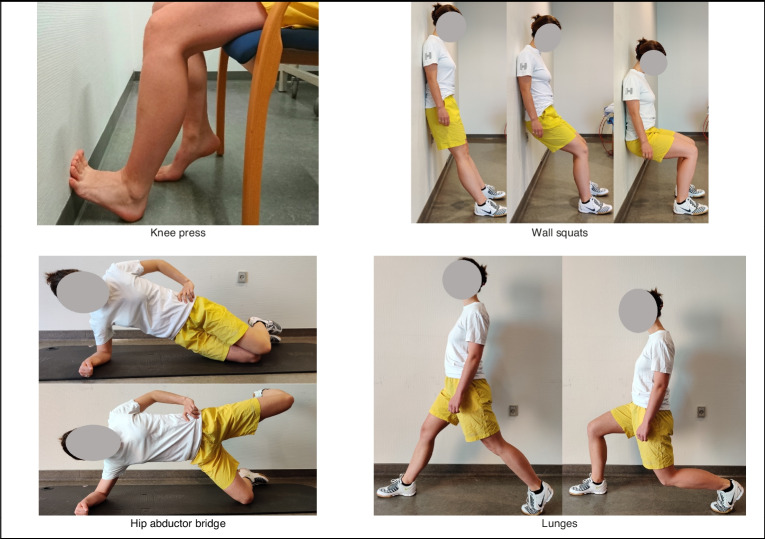
Depiction of hip- and knee exercises

*Exercises:* For targeting the insertion site of the patella tendon, the tibial tubercle, exercises at around 75° knee flexion will be performed, starting with the mild “knee-press” exercise during the first month, after which participants will be instructed to proceed to heavier weight-bearing wall-squats at appx 90° knee flexion, followed by unilateral lunges at approximately 125° knee flexion (Fig. 1). The tensile force on the patella tendon is approximately half that during a bodyweight squat compared to a lunge with high range of motion [38, 39]. The regression/progression of exercises will depend upon the pain experienced during and until the morning after performing the exercise – if pain has not exceeded NPRS 2, the standardized exercise-dose should be progressed. Alternatively, the exercise dose should be maintained or regressed until NPRS ≤2 is achieved. Besides knee-dominant exercises, the hip abductor bridge will be prescribed, with the same dose throughout months 0–3.

### Loading from sport and physical activity

Participants will be asked to take a complete break from weight-bearing sports and rigorous physical activity during the first month. After month 1, gradual exposure to sport will begin, using the same pain-model to progress or regress loading, as well as sport-specific advice from a physiotherapist based on load-markers such as intensity, frequency, duration. Once full or maximum-possible sports participation has been achieved, progression of load from rigorous physical activity will follow in the same manner. 

*Advice and education:* The leaflet and conversations during visits will contain information and advice regarding the following domains: 1) Aetiology 2) Pain management 3) Effects of exercise and knee loading activities 4) Prognosis 5) Self-management decision-making tools and addressing real-world challenges for the participant. From month 3 to 5, the participants will completely self-manage their condition and is encouraged to maintain some level of self-chosen exercise dose (advice of 1–3 sets of lunges and hip abductor bridge, 2–3 times weekly).

#### Comparator intervention: usual care

We have comprised a usual care treatment package encompassing the most common advice and modalities (see "Choice of comparators"). The treatment package includes a patient-aimed leaflet, which contains vignettes and elaborations of the multimodal approaches included in the usual care package, which will be implemented through four visits (at months 0, 1, 2, 3) with a physiotherapist (mirroring the plan of care of the experimental group). The modalities include: 1) Progressive balance and alignment exercises every other day on one of 6 progressing levels; 2) Stretching of the quadriceps muscle of the symptomatic leg(s), consisting of 2 daily sets of 30 seconds; 3) Use of a patella strap if participants find relief in its use, particularly during sport or physical activity; 4) Advice on cryotherapy and taping; and 5) Advice on load, pain, and prognosis.

### Progressive balance and alignment exercises

**Fig. 2:**
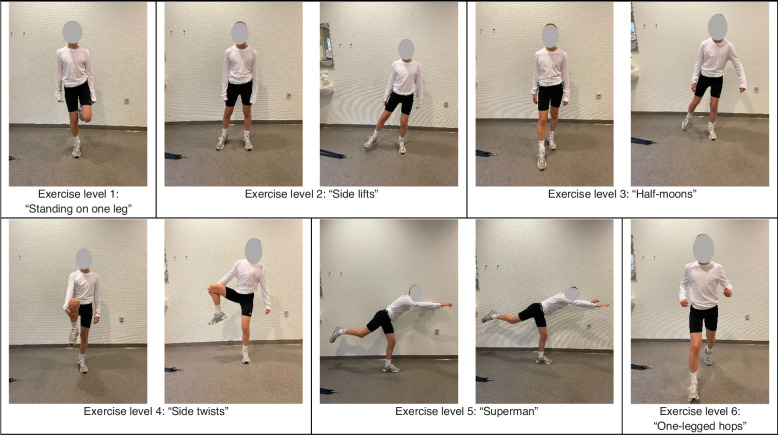
Depiction of progressive balance and alignment exercise levels

Participants will be performing one exercise incorporating balance and alignment every other day. The exercise can be one of 6 exercises on progressively more challenging levels (Fig. 2). The exercise levels has been sourced or inspired by sections from a leading sports rehabilitation textbook [13] and subsequently adjusted and refined with an experienced clinician utilizing this type of modality with Osgood-Schlatter patients (23 years practiced, seeing appx. 40 Osgood-Schlatter patients/year).

If a participant continuously experience pain during any of the exercises, the participant is advised to stop performing the exercises and regress to a previous pain-free level of exercise. If participants have bilateral Osgood-Schlatter, the exercises will be performed unilaterally, with each leg as the supporting leg interchangeably. For all the exercise levels, the participant must focus on stability/balance and hip-knee-foot alignment on the supporting/landing leg, defined as the symptomatic leg. When each exercise can be performed with a sufficient level of alignment and stability, the participant can progress to the next level. The participants will be instructed through their leaflet and visits with a physiotherapist on how to evaluated exercise quality and when to progress exercises. During each visit, the physiotherapists will also evaluate the current exercise level. All exercises levels are depicted in Fig. 2.

Alignment in this context is defined as the ability to

1. keep the pelvis horizontal in the coronal plane, neutral in the sagittal plane, and not twist around the center of mass in the axial plane
2. keep the knee from going into varus or valgus, and
3. keep the foot and midfoot arch from going into pronation or supination

Stability and balance in this context are defined as the ability to perform movement or holds without excessive perturbations, corrections, or otherwise inability to perform the movement with intended form or tempo.

- Level 1 exercise “Standing on one leg”: The exercise is performed by standing upright on one leg for 2 repetitions of 30 seconds.
- Level 2 exercise “Side lifts”: Standing upright on one leg, participants slowly and controlled lifts their contralateral straight sideways out in the coronal plane into hip abduction, and then back again, without the foot touching the ground. The exercise is performed for 2 sets of 10 repetitions in a slow controlled movement.
- Level 3 exercise “Half moons”: Standing upright on one leg, participants slowly and controlled tracks their contralateral foot just off the floor, drawing half-circles around themselves. The exercise is performed for 2 sets of 10 repetitions in a slow controlled movement.
- Level 4 exercise “Side twists”: Standing upright on one leg, the participant lifts the contralateral leg by grabbing their knee with one hand, tucks it toward their abdomen, and then rotates in the hip around their center of mass in the axial plane. Thereby the leg is slowly rotated to the side, all while keeping sufficient alignment on the supporting leg and sufficient stability and balance. The exercise is performed for 2 sets of 10 repetitions.
- Level 5 exercise “Standing Superman”: Standing upright on one leg with both arms flexed at the shoulder, and positioned straight in front them, they slowly and controlled lean forward with stretched arms while also lifting the other (asymptomatic) leg up behind themselves. The exercise is performed for 2 sets of 10 repetitions.
- Level 6 exercise: Participants perform hops by setting off on the asymptomatic leg and landing on the symptomatic leg, and alignment and balance/stability are then evaluated during landing. The hop is performed in three different directions; straight forward, sideways, and diagonally. After each hop, participants takes a step back to the starting position. The exercise is performed for 3 repetitions of 8 hops to each direction, totaling 24 hops.

#### Criteria for discontinuing or modifying allocated interventions

If scheduling or the availability of equipment (surface, chair) hinders the participant from following the exercise dosage, the treating physiotherapist will, together with the participant, try to amend the programme to better suit the preferences and context of the participant while still aiming for the correct dose and form in accordance with the original approach to the fullest extent possible.

#### Adherence strategy and monitoring

Making exercises enjoyable, social, and convenient, has been identified as the most likely facilitators to exercise adherence in adolescents with musculoskeletal pain [40]. In line with this, the exercises prescribed in both groups are designed to be performed with only little if any exercise-equipment and are time-efficient (1–20 minutes per day/every other day). We also encourage the participants to attend their regular sports team practices and perform the exercises in that environment, thereby gaining a social aspect of performing the exercises rather than potentially skipping practice altogether [41]. Through weekly text-based monitoring from week 0 to 22, participants are asked about their adherence to exercise the past week. Participants will be asked at visits at months 1, 2 and 3 about their current exercise dose and to demonstrate the exercises (month 1 and 3), which will then be rated by the observing therapist on a standardized form. Participants will be encouraged only to receive treatment as outlined in their allocated group. Concomitant treatment will not be course for exclusion, but will be recorded at visits months 1, 2, 3, and 5. Adherence to the 4-week break from weight-bearing sports and rigorous physical activity is monitored through accelerometry with a compliance-criteria of ≤15 min of VPA (vigorous physical activity) and ≤ 30 min of MPA (moderate physical activity) every day.

### Outcomes and harms

#### Assessment of outcomes

In Table 2 we have outlined the schedule of data collection for all outcomes. Weekly monitoring will be performed using a text-based service (SMS-track®, Esbjerg, Denmark) starting the following Monday after the baseline visit, and continue for 22 weeks. We will continue data collection irrespective of adherence to interventions. No biological samples will be collected. We have reported our outcomes in a prioritized order in addition to designations as primary and secondary (Table 3), which also reflects the order of hypothesis-testing, analyses, and intentional order of reporting, with the primary outcome, secondary outcomes no. 2–10, and adverse events (AEs), intended for the primary report [42].

**Table 2:** Study schedule

**Study phase**	Pre-allocation	Baseline	Intervention period				Primary endpoint	Clinical follow-up	Long-term follow-up^a^
**Timepoint**		Mo 0	Weekly	Mo 1	Mo 2	Mo 3	Mo 5	Mo 8	Mo 10, 12, 24, 48
**Enrollment procedures**									
Clinical visit		X		X		X	X	X	
At home	X		X		X				X
Phone screening	X								
Written information	X	X							
Verbal information	X	X							
Written consent		X							
Allocation		X							
**Clinical assessments**									
Clinical examination		X					X		
Peak Height Velocity		X				X	X	X	
Adverse events		X		X	X	X	X	X	
Previous & concomitant treatments		X		X	X	X	X	X	
**Clinical tests**									
Pressure-Pain threshold		X		X		X	X	X	
Knee extension strength		X		X		X	X	X	
Countermovement jump		X		X		X	X	X	
Modified Thomas test		X		X		X	X	X	
Anterior knee pain provocation test		X		X		X	X	X	
**Objective longitudinal measures**									
Physical activity (sensors)									
**Imaging**									
Ultrasound scanning		X				X	X	X	
**Patient-reported questionnaires**									
KOOS-child 4 subscales		X		X	X	X	X	X	X
Patient Acceptable Symptom State		X		X	X	X	X	X	X
Global rating of change				X	X	X	X	X	X
Sport & Physical activity		X	X	X	X	X	X	X	X
Pain history		X	X	X	X	X	X	X	X
EuroQol 5-Dimension Youth		X				X	X	X	X
Tampa Scale of Kinesiophobia		X				X	X		
Working Alliance Inventory						X			
Miscellaneous health^b^		X							
Pubertal stage (Tanner)		X				X	X		
Adherence to treatments			X	X		X	X	X	
**Intervention**									
Experimental or usual care treatment									
Complete self-management									

^*a*^= digital, ^*b*^= Sleep duration and problems, vitamin and supplement consumption. *PA* Physical activity*, QoL *Quality of Life

**Table 3:** Overview of outcome domains and prioritized hierarchy of outcomes

**Outcome domain**	**Specific outcome variables**				**Specific outcome variables**	
	**Primary outcome**					
Sport function	1. KOOS-child ‘Sport/play’ 0-100 subscale (7 items)					
	**Secondary outcomes**				**Secondary outcomes**	
Patient-acceptable Symptom-state	2. PASS question (Y/N)					
Knee-related Quality of Life	3. KOOS-child ‘Quality of Life’ 0-100 subscale (5 items)					
Pain intensity and frequency (Pain flares)	4. 4-week-average episodes of pain flares (≥4 on 0-10 NPRS) 5. Worst pain past week (0-10 NPRS)				11. KOOS-child question P1 on ‘Pain’ subscale (1-5 Likert scale) 12. Level of pain/discomfort (EQ-D5-Y 4)	
Participation in sports and physical activity	6. 4-week average hours of sports participation				13. 4-week average hours of MVPA 14. Satisfaction with extent of sports participation 15. Pre knee pain level of sports participation 16. Pre knee pain level of physical activity 17. Time to return to sport (week no.)	
Osgood-Schlatter morphology (ultrasound imaging)	7. Flaviis composite severity score				18. Tendinosis signs (thickening or hyperemia) 19. Infrapatellar bursitis signs (effusion or hyperemia 20. Hyperemia of the tibial tubercle ad modum Öhberg	
Pain during knee loading	8. Anterior Knee Pain Provocation test (0-10 NPRS)				21. Pain during knee extension test (0-10 NPRS) 22. Pressure-pain threshold at the tibial tubercle (kPa) 23. KOOS-child ‘Pain’ 0-100 subscale (8 items) 24. Known pain during manual palpation 25. Pain during countermovement jump (0-10 NPRS)	
Objective knee function					26. Maximal isometric knee extension strength (Nm/kg)27. Countermovement jump height (cm)28. Countermovement power (W)29. Knee extensor flexibility change (°)	
Global rating of change	9. 7-point Likert scale				30. Satisfaction with treatment (Y/N)	
Usual activities	10. Patient-specific function scale (NRS 0-10)				31. Problems with usual activities (EQ-D5-Y 3)	
Pain beliefs					32. Kinesiophobia (Tampa Scale of Kinesiophobia, 17 items)	
Health					33. Self-rated health (EQ-D5-Y 0-100 VAS)	
	**Safety outcomes**					
	1. Any adverse events					

*KOOS child* Knee injury and Osteoarthritis Outcome Score – Child, *PASS *Patient Acceptable Symptom State, *NPRS *Numerical Pain Rating Scale, *EQ-D5-Y* Euro-Qol 5 Dimensions - Youth, *MVPA *Moderate-to-Vigorous Physical Activity, *VAS* Visual Analogue Scale

#### Primary outcome measure

The primary between-group difference will be evaluated using the self-reported outcome measure KOOS-child (Knee injury and Osteoarthritis Outcome Score – Child), designed specifically for adolescents aged 10–18 years experiencing knee problems [43]. The questions are answered on 5-point Likert scales and pertains to the prior week. The scoring of each subscale is normalized to a 0–100 score, 0 being extreme symptoms and 100 being no symptoms. Four subscales (‘Symptoms’, ‘Pain’, Sport/play’, and ‘Quality of Life’) will be recorded and presented, and one omitted (‘Activities of Daily Living’ due to low responsiveness [44, 45]). The subscale ‘Sport/play’ will be prioritized and used as primary outcome, based on its ability to capture self-reported difficulties in performing sports-related activities and movements, and also due to its alignment with study aims, responsiveness in the target population [44, 45], and preferences from patient representatives. The ‘Sport/play’ subscale will thus provide properties for sample size calculation. The KOOS-child has low detectable change on the group level (1–3 points, *n* = 70) and substantial/near-perfect test-retest reliability (ICC 0.78–91) [43]. For the KOOS-child ‘Sport/play’ subscale, the smallest detectable group-level change is 3 points and the standard error of measurement are 8 points [43].

#### Secondary outcomes

Remaining outcomes are outlined in Table 3, and includes self-reported measures, clinical tests, and ultrasound imaging.

#### Adverse events and harms

In our published cohort-study [17] and during pilot-testing of the intervention in the clinic [44], we have not observed any AEs related to undergoing the treatment, imaging or clinical tests. As part of the accelerometry-based monitoring of physical activity, adhesive patches are used to fix the sensor to the skin. Skin irritation due to sensor-patches was observed in four participants during piloting. The manufacturer has since provided adhesives made of a new FDA-approved breathable woven material to mitigate this issue. Adverse events during trial-participation will be graded according to the Common Terminology Criteria for Adverse Events (CTCAE) grading (grade 1–5) [46]. To probe for potential any suspected harms or AEs, participants will be asked during all clinical visits and the telephone consultation at month 2, through open-ended questioning about any new symptoms or illnesses, accidents, reasons for care-seeking. In addition, serious (grade 3–5) unexpected side effects or AEs will be reported to the Capital Regional Ethics Committee in Denmark within 7 days after the study director has become aware of the incident. All serious adverse events will be assessed by the study director and the Medical Advisor (PH) for possible relations with the assessments and/or intervention.

### Study schedule and post-trial care

**Fig. 3:**
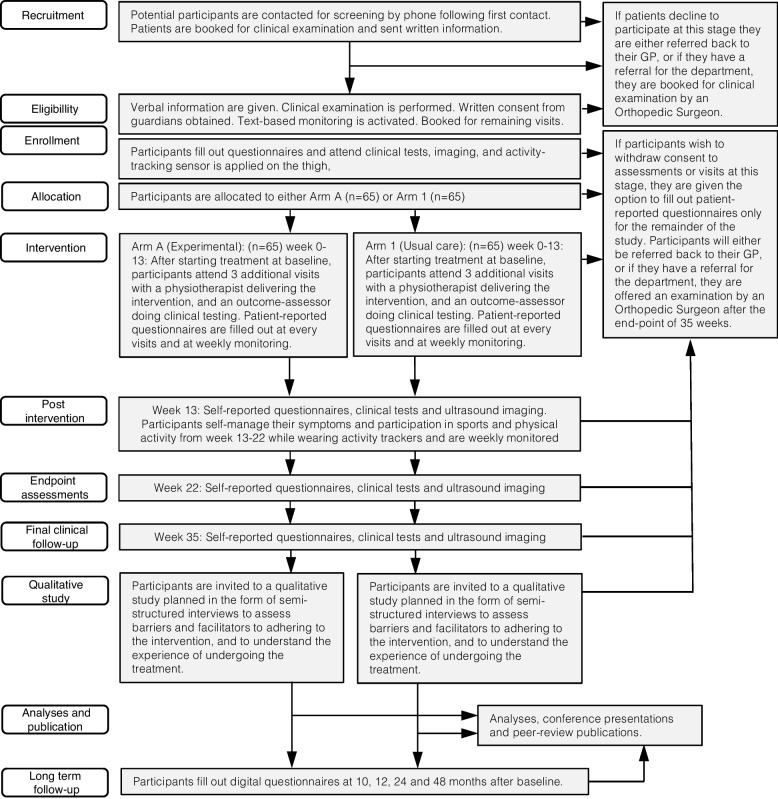
Study flow

After baseline, participants have visits at months 1, 2, 3, 5 and 8, and electronical long-term follow-up after 10, 12, 24 and 48 months (Fig. 3).

The primary and secondary fixed endpoints are chosen based on the fact that our previous cohort had trajectories of pain, knee-function, and sports participation that were not fully recovered at 12 weeks [17], and that some participants had not progressed fully through the exercise regime. Thus, we decided to add a period of complete self-management by adding 2 months to the timeline before the primary endpoint. We have found this alteration to be feasible in clinical practice [44]. No participants will receive reimbursement for their travel expenses related to participation in the study, nor will participants be offered compensation of any kind for their participation. No post-trial treatment is planned, but will be implemented on a case by case evaluation. During treatment at Hvidovre Hospital, the participants will be covered under the Danish Patient Compensation Act (LBK no 995 of 14/06/2018, chapter 3 §19) (In Danish: Patienterstatningen), which is a scheme that deals with compensation claims of patients treated in the public health system in Denmark who has sustained an unintended or unexpected injury or harm.

### Inclusion criteria

The diagnosis of Osgood-Schlatter and eligibility for trial participants will be made according to the following criteria: 1) Pain or swelling of the tibial tubercle for ≥6 weeks with a primary insidious onset, which is provoked by at least 2 of the following positions or activities; prolonged sitting or kneeling, squatting, running, hopping/jumping, stair walking, or during multidirectional sports; 2) Tenderness on palpation of the tibial tubercle or pain during resisted isometric knee extensions. Adolescents aged 10–16 fulfilling these criteria at enrollment and confirm when asked during clinical examination that they either 1) “*have markedly reduced sports participation”,* or 2) *“are severely affected by pain during participation during the past (representative) 6 weeks?”,* will be eligible for inclusion. Any other primary pathology or complaints from other structures of the knee will disqualify the participant from inclusion but will be allowed providing that primary complaints during the preceding ≥6 weeks are from the tibial tubercle. Previous fractures or avulsions of the tibial tubercle will disqualify patients.

### Sample size estimation

In the absence of minimal important change thresholds for KOOS-child ‘Sport/play’ scale, specific in terms of diagnosis, severity, intervention, and length of follow [47], we have performed a clinical case series accounting for these factors, providing specific and relevant estimates of change on the different KOOS-Child subscales. In this clinical case series, 16 out of 33 patients met eligible criteria for inclusion in the current trial at their baseline visit, received the intervention in question, and attended follow-up at 6 months. The subscales most affected at baseline which also showed high responsiveness to change were ‘QoL’ and ‘Sport/play’. Furthermore, patients preferred the ‘Sport/play’ subscale, and we therefore decided to use the ‘Sport/play’ subscale as the primary outcome measure. We will utilize a 9 point difference as a threshold for determining superiority. We consider this to be a meaningful longitudinal change, a relevant between-group difference, and is within the recommended range of 8–10 points (heartbeat-med.com/resources/knee-injury-and-osteoarthritis-outcome-score-koos). All 16 patients from the case series reported their global rating of improvement on a 7-point Likert scale ranging from ‘much worse (7) ‘to ‘much better (1)‘. Patients who reported ‘much better (1)’ or ‘better (2)’ had a mean improvement on KOOS-child ‘sport/play’ subscale of 12.7 ± 16 points, whereas patients reporting less improvement (‘little’ improvement or ‘no change’) only had a 3.6 point increase. Thus, the 9 point difference also distinguished patients in our case series who reported meaningful improvements, from those who did not. To detect this 9 point difference with a standard deviation of 16 points, at an α-level (type I/false positive error rate) of 5 and 90% power (β-1, or probability of avoiding type II/false negative error rate), *n* = 55 participants per group would be needed based on an independent one-sided t-test (R 4.0.2, Foundation for Statistical Computing, Vienna, Austria; RStudio 1.0.153, power.t.test package). To account for a potential 15% dropout rate [37] a total of 130 participants will be included. This will correspond to a 0.56 Cohens d ‘medium’ effect size at 90% power. The smallest effect size reliably detectable will thus be > 0.482 (Cohens d ‘small’ effect size) at ≥80% power (Jamovi 1.2.25, jpower module), surpassing the trivial (< 0.2) and small (> 0.2) effect size thresholds. 

### Group allocation

#### Allocation concealment

The sequence will be implemented using sequentially numbered sealed opaque envelopes, sealed and prepared by a person not otherwise involved in the trial. During the trial, only the person responsible for including participants will unseal envelopes when a participant is included in the trial.

#### Sequence generation and implementation of group allocation

Participants will be allocated to either group with a 1:1 allocation ratio. To perform adequate sequence generation, we will use The Robust Randomization App [48] (RRApp v3.0.1, https://clinicalresearch-apps.shinyapps.io/rrapp/) for a computer-generated sequence in random sized blocks with no stratifications, extracted by a person not otherwise involved in the trial. During the study period, all staff involved in including participants, treatment, data collection, and analysis will be blinded to randomization sequence and block size.

### Recruitment and participant retention

Participants will be recruited through a combination of convenience sampling from the uptake area of the Capital Region of Copenhagen, Denmark (1.8 M inhabitants), through two different approaches; 1) postings to our website encouraging parents of adolescents with confirmed Osgood-Schlatter or anterior knee pain below the knee to contact the study director, which will also be shared with sports clubs in the uptake area through our organizational network and social media; and 2) patients referred to specialized outpatient clinic at the Orthopedic Department. Based on historical patient flow and past studies in this population, the planned recruitment rate is expected to be around 10–15 participants per month. Thus, inclusion is expected to last no more than 2 years, from January 1st 2022 to December 31st 2023.

### Blinding

To yield valid results from the trial and in lieu of clinical equipoise in usual care (see Choice of comparators section), we will blind participants to the superiority hypothesis and specific contents of the intervention they are not receiving. This will be done by providing minimal information to participants about the contents of either intervention until after group allocation. The written and verbal information prior to inclusions will state that the two groups both contain first line treatment modalities such as different advice and exercises and that it is unknown if any treatment is superior. Information on which of the two treatments are hypothesized to be superior, will be withheld. This blinding aspect will reduce the risk of performance bias. To further minimize performance- and verification bias, the intervention personnel will not be aware which, or if any, of the two treatments is the experimental or the comparator, or if the trial is investigating superiority/non-inferiority/equivalence. Intervention personnel will need to engage participants in conversations about their current pain and symptoms, and will therefore not be blinded to outcomes as such. Outcome assessors and the statistician performing analysis will be blinded to group allocation. As the study director and the Medical Advisor is unblinded to group allocation, unblinding is not expected to be necessary during the trial.

### Data management and confidentially

The study director will manage and curate data in collaboration with the blinded statistician (TK). Written consent forms and other hardcopy data will be stored in locked steel cabinets in a locked room and will be stored for 3 years after completion of the long-term follow-up of the study. All outcome data, besides text-messages, ultrasound images, and sensor-data, will be entered into REDCap. We will keep standard confidential electronic health records in accordance with local laws and healthcare regulations. In addition, our plans (and sub-contractors) for use and handling of patient-data have been reviewed and approved by the Capital Region Data Protection Agency (P-2021-818) after the protocol was approved by the Capital Region Committee on Health Research Ethics (H-21028912). We plan for only the study director to hold access to the de-identifier key. As per usual care, information regarding clinical findings, treatment plans, and delivery, will be noted in the participants regular electronic medical records to support potential post-trial care. All statistical code and fully anonymized dataset will be shared in an open-access repository once all planned publications are accepted or published as pre-print [49, 50].

### Statistical analysis

A statistical analysis plan is embedded in the full protocol (NCT05174182). Analyses will be performed by a statistician (TK) blinded to group allocation. Change scores for KOOS-child ‘Sport/play’ score (△KOOS-child) from baseline to month 5 will be calculated for all participants. We will fit a linear regression model for △KOOS-child as the outcome variable, and group allocation as the predictor variable. Potential covariates are described in Table 4.

**Table 4:** Proposed moderators and mediators

Variables	Measurement	Justification
**Potential moderators/covariates**		
Growth velocity_w0_	Offset from predicted age at peak height velocity in years, calculated from clinically measured anthropometrics: sitting height, bodyweight, total stature, biological age [51]. Participants will be categorized into pre- (<− 1.0 years offset), circa- (− 1.0 to + 0.5 years offset), and post-PHV (> + 0.5 years offset), corrected for timing of the primary endpoint, resulting in a 5 month subtraction.	Overuse knee injuries, as well as injuries to the growth plate, in sports active adolescents are higher during peak height velocity and the year leading up to this point [52–56], which is thought to be primarily due to vulnerable growth-related conditions, such as Osgood-Schlatter [52]. This has been supported by data showing higher growth velocity for Osgood-Schlatter patients than controls [57].
Growth timing_w0_	Based on algorithms for calculating anticipated age of peak height velocity which incorporates data on parents adult stature, we will classify participants as early maturers, average maturers, and late maturers (girls: < 10.94, 10.94–12.94, and > 12.94 years, respectively; boys: < 12.64, 12.64–14.64, and > 14.64 years, respectively) [58, 59].	Reaching skeletal maturity either late or on average is a risk factor for developing Osgood-Schlatter compared to early maturation [2, 60].
Fear of movement_w13_	Fear of movement will be captured by participants filling in the 17 items Tampa Scale of Kinesiophobia, each item scored on a 4-point likert scale with a score ranging from 0 to 68 [61, 62].	Avoidance behavior might be related to a too apprehensive approach to gradual exposure and exercise therapy resulting in the patient not achieving a higher degree of participation in sports and physical activity. Osgood-Schlatter patients exhibits a high degree of kinesiophobia [44].
Pain intensity_w0_	Self-reported “worst pain past week” on the 0–10 NPRS.	Pain intensity has shown to be related to a worse prognosis for adolescents with anterior knee pain [63]
Pain frequency_w0_	Self-reported on the P1 question of the KOOS-child Pain subscale on frequency of experience knee pain, answered on a 0–4 ordinal scale ranging from “Never” to “All the time” [43].	Pain frequency have shown to be related to a worse prognosis for adolescents with anterior knee pain [63, 64].
Treatment expectations_w0_	Self-reported through the question on change in “my ability to self-manage my knee pain” answered on a 1–4 likert scale from ‘worse’, ‘no difference’, ‘little improvement’, to ‘large improvement’.	Treatment expectations have been shown to moderate outcomes in trials of several different musculoskeletal conditions and chronic pain conditions [65–69].
Tibial tubercle maturation_w0_	Rated by sonographers *ad modum* Sailly [70, 71] on a 1–4 scale depending on features of cartilage, potential secondary ossification center, tendon, and the infrapatellar bursa.	The level of maturation of the apophysis has been shown to be related to the prevalence of Osgood-Schlatter symptoms with early (exhibiting no metaphysis-physis junction or apophyseal attachment of the patella tendon) and late stages (denoting full unification of metaphysis-physis junction and matured attached of the patella tendon) being low-risk stages, and the intermediate stages (exhibiting open metaphysis-physis junction, apophyseal attachment of the patella tendon, active ossification center) have a higher association to symptoms [70, 72, 73]
Severity_w0_	Rated by sonographers *ad modum* Flaviis [73] from ‘cartilage attachment’ to ‘mature attachment’ on a 1–4 scale.	Severity on the Flaviis scale has been associated with a worse prognosis [9, 73].

**Table 5:** (Table 5)

Author contribution							
	KK	PH	KT	JL	MR	MC	TK
Conceptualization							
Funding acquisition							
Supervision							
Resources							
Draft of protocol							
Review and edit of protocol							
Methods: Trial design							
Methods: Analyses plan							
Methods: Imaging							
Methods: Interventions							
Methods: Outcomes							

The linear model will be evaluated for linearity, multicollinearity, homogeneity of variance, distortion of outliers, homoskedasticity, and distribution of residuals using plots and scripts for model-checks. If these model assumptions are not met, non-parametric bootstrap estimation and tests will be used instead. To examine the effect of adherence we will perform sensitivity analyses of subgroups according to pre-specified compliance criteria. No interim analyses or stopping rules are planned, due to very low safety concerns and to preserve statistical power. Assuming that data will be missing at random, multiple imputations using chain equations will be used to handle missing data. Imputation models for missing variables will be fitted using linear, logistic or polytomous regression models. All available variables will be included in the imputation models, unless a specific reason is given for exclusion. Imputation will only be performed for variables included in the analysis. Multiple imputation will be done using R-package mice [74] Table 5.

### Data sharing, authorship, and dissemination

All members of the study group will be invited as co-authors on the specific publications according to the International Committee of Medical Journal Editors (ICMJE) recommendations and The Danish Code of Conduct for Research Integrity codec [75, 76]. All findings and results are planned to be published in international peer-reviewed scientific journals. The results will be posted to ClinicalTrials.gov once the results have been published. The results will be published regardless of positive, negative, or inconclusive findings. No trial conduct audit is planned. Kasper Krommes and Per Hölmich will enforce publications as first and senior authors respectively, unless other publication-specific contributions warrants.

### Protocol amendments and versions

The protocol have been published in several version. Version 1.0 was approved by ethics review board. Version 1.1 and 1.2 included all SPIRIT items, the embedded SAP, and was posted to ClinicalTrials.gov before recruitment started without details of the interventions, in order to preserve blinding of potential participants and staff. The subsequent versions (v1.3 01-MAR-2023 and v1.4 05-APR-2024) had updates on pilot-results, qualitative study, mediation analysis, and details on the interventions as blinding was no longer an issue. This version (v1.5) is formatted for journal publication.

### Trial audit, steering committee, and contact details

The Sports Orthopedic Research Center – Copenhagen (SORC-C), specifically PhD-fellow Kasper Krommes (Study Director), Professor Per Hölmich (Main Supervisor and Medical Advisor), and Professor Kristian Thorborg (Co-supervisor) at the Department of Orthopedic Surgery at Amager-Hvidovre Hospital has initiated and will manage the trial. Together, they also form the steering- and writing committee, which will oversee the trial and assume stewardship of the data. No specific data monitoring committee is convened.

## Data Availability

Protocol-related materials are available at open-access repository (doi.org/10.6084/m9.figshare.c.5730008.v1).
